# Curcumin alleviates macrophage activation and lung inflammation induced by influenza virus infection through inhibiting the NF‐κB signaling pathway

**DOI:** 10.1111/irv.12459

**Published:** 2017-07-11

**Authors:** Yiming Xu, Ling Liu

**Affiliations:** ^1^ Department of Respiration Medicine The Affiliated Wuxi Second People's Hospital of Nanjing Medical University Wuxi China

**Keywords:** acute lung injury, curcumin, inflammation, Influenza A viruses (IAV), nuclear factor kappa‐light‐chain‐enhancer of activated B cells (NF‐κB)

## Abstract

**Background:**

Influenza A viruses (IAV) result in severe public health problems with worldwide each year. Overresponse of immune system to IAV infection leads to complications, and ultimately causing morbidity and mortality.

**Objective:**

Curcumin has been reported to have anti‐inflammatory ability. However, its molecular mechanism in immune responses remains unclear.

**Methods:**

We detected the pro‐inflammatory cytokine secretion and nuclear factor kappa‐light‐chain‐enhancer of activated B cell (NF‐κB)‐related protein expression in human macrophages or mice infected by IAV with or without curcumin treatment.

**Results:**

We found that the IAV infection caused a dramatic enhancement of pro‐inflammatory cytokine productions of human macrophages and mice immune cells. However, curcumin treatment after IAV infection downregulated these cytokines production in a dose‐dependent manner. Moreover, the NF‐κB has been activated in human macrophages after IAV infection, while administration of curcumin inhibited NF‐κB signaling pathway via promoting the expression of nuclear factor of kappa light polypeptide gene enhancer in B‐cells inhibitor, alpha (IκBα), and inhibiting the translocation of p65 from cytoplasm to nucleus.

**Conclusions:**

In summary, IAV infection could result in the inflammatory responses of immune cells, especially macrophages. Curcumin has the therapeutic potentials to relieve these inflammatory responses through inhibiting the NF‐κB signaling pathway.

## 1 INTRODUCTION

Influenza A viruses (IAV) cause severe public health problems with worldwide each year, among which the acute respiratory distress syndrome (ARDS) or acute lung injury (ALI) result in the majority of influenza pneumonia associated death.^1^, ^2^ It is recorded that in 1918, approximately 50 million people lose their lives worldwide because of reoccurring pandemics.^3^ The host target cells, such as epithelial cells, have an essential role in responding to the IAV infection. In addition to this, the responses of immune cells, especially the macrophages are also important.^4^, ^5^

Airway macrophages (AMs) are among the first line of body defense upon the infection of IAV. The AMs have the ability to phagocytose virions and IAV‐infected cell, and its infection symbolizes an early stage of virus recognition by the innate immune system.^6^, ^7^ In addition to the early stage functions, macrophages were also found to modulate both the innate and adaptive immune responses at the late stages,^8^ as well as to be essential antigen presenting cells.^9^ The importance of its responses can be demonstrated by depletion of alveolar macrophages before IAV infection, which caused increased morbidity, mortality, and symptom severity.^10^, ^11^ However, the overresponse of macrophages to IAV infection also results in pathogenesis. It has been reported that the tissue damage caused by macrophages contributes to the following bacterial infections, and ultimately causing morbidity and mortality.^12^, ^13^

Inflammation is an adaptive physiological response following infection and tissue injuries. It is the results of series immune responses, and also leads to varieties of morbidities. Extensive studies showed that inflammation was correlated with altered signaling pathways, with enhanced levels of inflammatory markers, free radicals, and lipid peroxides. Inflammation has been hypothesized to play an essential role in competing infection and in wound healing.^14^ Thus, restriction of macrophage inflammatory responses to IAV infection has important clinical significance.

Curcumin, the major curcuminoid of the turmeric, has multiple health‐promoting biological activities.^15^ Curcumin was first used as an anti‐inflammatory agent since ancient times. Because of its anti‐apoptotic, anti‐oxidative, and anti‐inflammatory abilities, curcumin was then widely used for preventing or treating a variety of diseases other than inflammation.^16^, ^17^ The anti‐inflammatory properties of curcumin have been well studied. For example, it was reported that curcumin inhibited diverse signaling pathways, including NF‐ĸB, cyclinD1, Cyclooxygenase‐2 (COX‐2), tumor necrosis factor alpha (TNF‐α), signal transducer, and activator of transcription (STAT).^18^ Jin et al.^19^ found that curcumin inhibits interleukin 6 (IL‐6) and TNF‐α expression in lipopolysaccharide (LPS)‐induced BV2 microglia cell. Cho and colleagues revealed that in TNF‐α‐treated HaCaT cells, curcumin reduced interleukin‐1β (IL‐1β) and IL‐6 production via inhibiting NF‐ĸB and mitogen‐activated protein kinase (MAPK) signaling pathways.^20^ In addition, the analogs of curcumin, such as B06 and C66, also have anti‐inflammatory ability through inhibiting NO and TNF‐α production, and reducing mRNA expression of cytokines such as COX‐2, IL‐12, IL‐6, TNF‐κ, and IL‐1β.^21^, ^22^ Even the anti‐inflammatory ability of curcumin was well investigated, its anti‐IAV function studies are very limited. Umar et al.^23^ utilized turkeys to study the possible effects of curcumin on immuneresponse and pathogenesis of H9N2 avian influenza virus (AIV). They found that in cooperation with thymoquinone, curcumin could be able to dramatically improve the immune responses and restrain the pathogenesis of influenza viruses in turkeys.

In our present study, we aim to investigate whether curcumin can relieve the lung inflammation caused by IAV infection, and its possible mechanisms and therapeutic potential.

## 2 METHODS

### 2.1 Preparation and culture of human macrophages

The human mononuclear cells were separated from the peripheral blood of healthy blood donors by utilizing Ficoll‐Paque (Amersham Biosciences, Sunnyvale, CA, USA). From the peripheral mononuclear cells, the CD14^+^ cells were isolated using VarioMACS™ technique and anti‐CD14 microbeads (Miltenyi Biotec, Bergisch Gladbach, Germany), through high‐gradient magnetic sorting. Macrophages were then induced from CD14^+^ monocytes for 6 days, which were cultured in complete RPMI‐1640 medium with 10 ng/ml human macrophage colony‐stimulating factor (M‐CSF) (R&D System, Minneapolis, MN, USA). Curcumin (Sigma, St. Louis, MO, USA) was dissolved in ethanol at a concentration of 10 mmol/L as stock solution. It was further diluted in phosphate‐buffered saline (PBS, vehicle) for use.

### 2.2 Virus preparation and macrophages infection

IAV strain A/Taiwan/3530/2001 (H1N1) was used to infect human macrophages, while the A/Puerto Rico/8/34 (H1N1; PR/8) was used for murine infection. These IAV strains were grown in Madin‐Darby canine kidney cells (MDCK, ATCC^®^CCL‐34™) based on the standard protocol. Virus titer was detected according to the plaque assay on confluent MDCK cells. Macrophages were washed with phosphate‐buffered saline (PBS) and re‐suspended in 100 μL of serum‐free RPMI 1640 with IAV at indicated multiplicity of infection (MOI=1) for 1 hour at 37°C. The cells were collected by centrifugation, and the supernatant was removed. Then cells were cultured by RPMI 1640 supplemented with 10% fetal calf serum (FCS) at 37°C.

### 2.3 Measurements of cytokine levels by enzyme‐linked immunosorbent assay (ELISA)

ELISA kits (R&D Systems) were used for detection of TNF‐α, interferon‐alpha (IFN‐α), and IL‐6 levels in cell culture supernatant and mouse bronchoalveolar lavage fluid (BALF) according to the manufacturer's instructions.

### 2.4 Western blots

Cells were gently re‐suspended in 500 μL 1×Hypotonic Buffer by pipetting up and down several times, which was incubated on ice for 15 minutes. 25 μL detergent (10% NP40) was added and vortexed for 10 seconds at highest setting. The homogenate was centrifuged for 10 minutes at 1600 *g* at 4°C. This supernatant contains the cytoplasmic fraction. The pellet is the nuclear fraction. Nuclear pellet was re‐suspended in 50 μL complete Cell Extraction Buffer for 30 minutes on ice with vortexing at 10‐minute intervals and centrifuging for 30 minutes at 14 000 *g* at 4°C. The supernatant (nuclear fraction) was transferred to a clean microcentrifuge tube. Protein concentration was measured by the Bradford protein assay.

Denatured proteins were separated by 10% sodium dodecyl sulfate‐polyacrylamide gel electrophoresis (SDS‐PAGE) and then transferred onto polyvinylidene difluoride (PVDF) membranes (Millipore, Billerica, MA, USA). The transferred membranes were blocked with 5% milk in tris‐buffered saline Tween 20 (TBST) for 1 h at room temperature and then incubated with cyclin G2 (CCNG2) antibody (Cell Signaling Technology, Danvers, MA, USA) overnight at 4°C. The membranes were washed for three times with TBST, and incubated with horseradish peroxidase (HRP)‐conjugated secondary antibodies in TBST for 1 hour at room temperature. After three times washing with TBST, the immunoblotting signals were detected via the chemiluminescence method (ECL, Millipore).

### 2.5 Animal treatment

The mice were provided by the Animal Center of Nanjing Medical University and were randomly set into seven groups. Eight mice were allocated in each experimental group. Mice from group one were without any treatment, while those from the other six group were exposed by intratracheal route to 2.5 LD50 of Influenza A/Puerto Rico/8/34 (H1N1; PR/8) virus. Mice were challenged with IAV daily for consecutive 4 days. On each virus treatment day, one hour after the exposure to virus, curcumin with different dose (ranging from 0 to 400 mg/kg) was administrated to mice of different group by intraperitoneal injection. Animal studies were in accordance with the standard protocols approved by the ethic committee of The Affiliated Wuxi Second People's Hospital of Nanjing Medical University (approval number #WXSPH207).

### 2.6 Analysis of inflammatory cells in BAL fluid

PBS was slowly implanted into the lungs of mice and then a cannula that had been inserted into the trachea was used to withdraw the liquid. Cells were harvested by centrifugation at 500 *g* for 7 minutes, and the supernatants were collected and stored at −80°C until needed. Cells were counted by hemocytometer and stained with a panel of fluorescence‐conjugated antibodies (BD Biosciences, Franklin Lakes, NJ, USA). Fluorescently labeled cells were analyzed by Calibur™ flow cytometer (BD Biosciences), and the data were analyzed by CellQuest™software (BD Biosciences).

### 2.7 Statistical analyzes

All the experiments data were statistically analyzed and performed using Prism software package (GraphPad Software, Version 5.00, San Diego, CA, USA). All the values are expressed as mean±SD. The significance of differences between groups was analyzed by the one or two‐way analysis of variance (ANOVA) followed by the Tukey‐Kramer test.

## 3 RESULTS

### 3.1 Curcumin has no effects on macrophage viability and the percentage of IAV‐infected human macrophages

**Figure 1:**
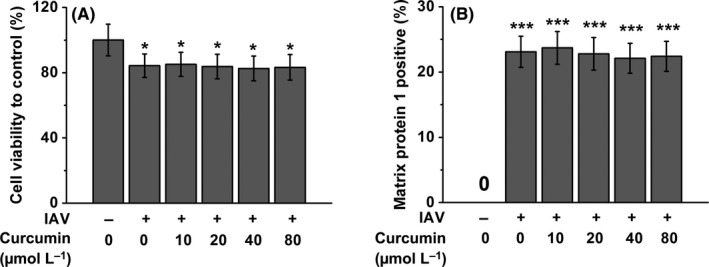
Curcumin treatment has no obvious effects on cell viability (A) and percentage of influenza A viruses (IAV) infection (B) in human macrophages. Cells were exposed to IAV for 1 hour and treated with curcumin at indicated concentrations (10, 20, 40, or 80 μmol/L) for another 24 hour before examination. Cell viability was determined by neutral red uptake assay. Percentage of IAV‐infected macrophages was determined by staining with IAV matrix protein 1, and then analyzed by FACS. Data are mean±SD *P<.05, *** *P*<.001 vs control (first column)

As it has been known that macrophages play an essential role in IAV infection,^24^ and curcumin can modulate the macrophage inflammatory responses,^23^, ^25^ we first ask whether curcumin has impact on macrophage viability under the infection of IAV. To address this question, we used curcumin of different concentration to treat IAV‐infected human macrophages. As shown in Figure 1A, the infection of IAV induced slight decrease of macrophage survival rate, and the administration of curcumin at different concentrations (ranging from 10 to 80 μmol/L) cannot rescue this IAV‐induced cell viability decrease of macrophages. To investigate whether curcumin treatment affects IAV infectivity of macrophages, we measured the intracellular IAV matrix protein 1 in macrophages after IAV infection. We found the treatment of curcumin failed to obviously impact the percentage of virus‐infected macrophages Figure 1B.

### 3.2 Curcumin treatment attenuates cytokine secretion in IAV‐infected human macrophages

**Figure 2:**
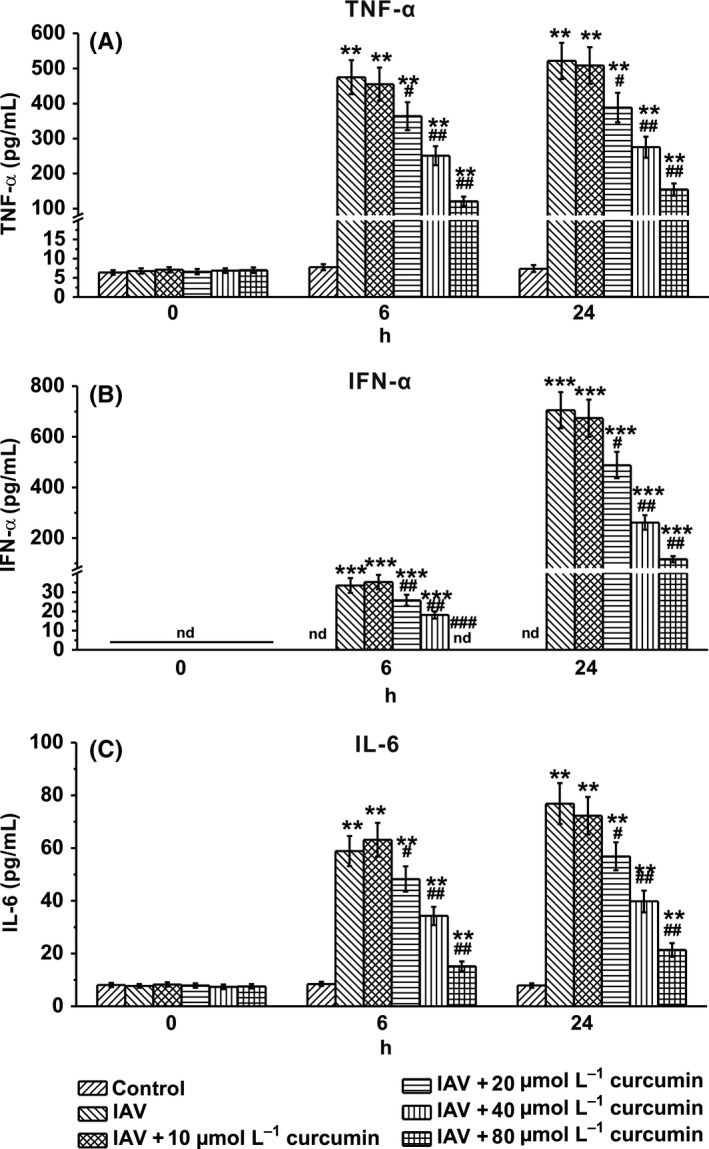
Curcumin treatment attenuates cytokine secretion in influenza A viruses (IAV)‐infected human macrophages at indicated time point post‐infection. Human macrophages were exposed to IAV for 1 h. Curcumin was presented in the culture medium until examination. Cytokines including TNF‐α (A), IFN‐α (B), and IL‐6 (C) were measured by corresponding enzyme‐linked immunosorbent assay (ELISA). Data are mean±S.D. **P<.01, ***P<.001 vs control; #*P*<.05, ##*P*<.01, ###*P*<.001 vs IAV group. nd means not detectable

To further investigate the effect of curcumin on IAV‐infected macrophages, we determined the secretion of the pro‐inflammatory cytokines TNF‐α, IFN‐α, and IL‐6, which contribute to the IAV‐induced inflammation. We found that at the time point of 6‐ and 24‐hour post‐infection of IAV, secretion of these cytokines showed a curcumin dose‐dependent decrease, compared with the control group which received no curcumin treatment (Figures 2A‐C). These results indicated that curcumin might have an anti‐IAV ability.

### 3.3 Curcumin suppresses cytokine production in IAV‐infected macrophages via downregulation of NF‐κB signaling pathway

**Figure 3:**
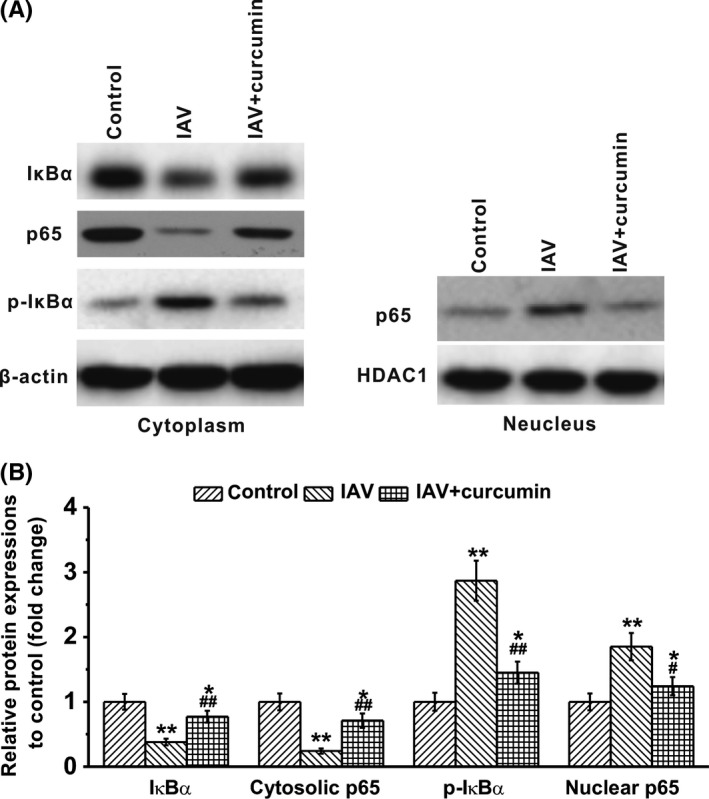
Effects of curcumin treatment (80 μmol/L) on NF‐κB signaling pathway in influenza A viruses (IAV)‐infected human macrophages at 6‐hour post‐infection. Human macrophages were exposed to IAV for 1 hour and then were harvested for Western blotting analysis at 6‐hour post‐infection. Curcumin was presented in the culture medium until examination. Expressions of NF‐κB signaling pathway‐related proteins, including IκBα, cytosolic p65, p‐IκBα, and nuclear p65 were analyzed by Western blotting. β‐actin was employed as a loading control. Data are mean±SD *P<.05, **P<.01 vs control; #*P*<.05, ##*P*<.01 vs IAV group

It has been reported that before IAV stimulation, p38 MAPK and extracellular signal‐regulated kinases (ERK) were constitutively expressed and activated in macrophages. After IAV infection, the NF‐κB inhibitor IκBα could be phosphorylated and degraded.^26^ To further investigate the mechanisms involved in curcumin‐regulated cytokine production of IAV‐infected macrophages, we conducted Western blotting to detect the expression of associated proteins upon IAV infection and curcumin treatment. We found that IAV infection induced a decrease of cytosolic IκBα level, and an enhancement of phosphorylated IκBα (Figure 3A and B), indicating an activation of NF‐κB. However, administration of curcumin on IAV‐infected macrophages reversed this phenomenon, resulting in the increase in cytosolic IκBα and the decrease in its phosphorylation in the cytoplasm.

P65 is a protein involved in NF‐κB heterodimer formation, nuclear translocation, and activation.^27^ It showed that IAV infection of macrophages leads to a significant decrease in cytosolic p65, while the levels of nuclear p65 increased under such condition. However, when the cells were treated with curcumin, we detected that both cytosolic p65 and nuclear p65 returned back to the resting levels (Figure 3A and B). Our results demonstrated that upon IAV infection, macrophages might be activated to secret inflammatory cytokines through activation of NF‐κB signaling pathway, while the antivirus ability of curcumin might depend on inhibition of NF‐κB in macrophages.

### 3.4 Curcumin inhibited the in vivo expanding of immune cells and cytokine production in mice BAL fluid upon IAV infection

**Figure 4:**
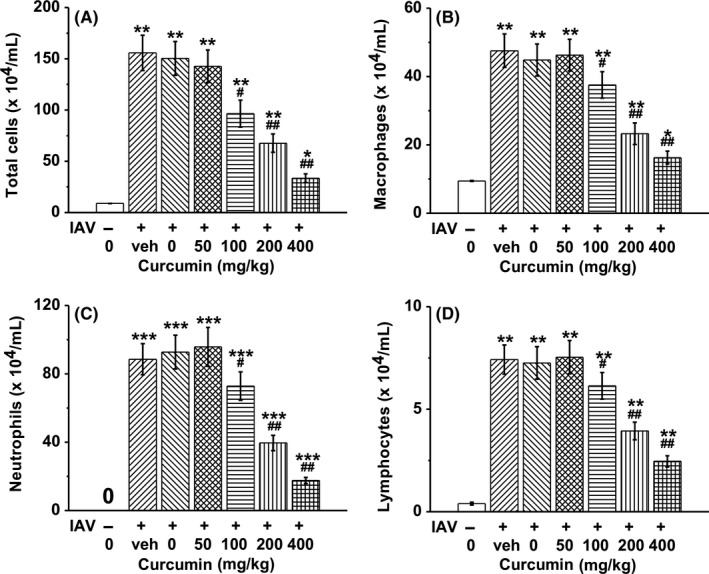
Effects of curcumin treatment on immune cell profiles in BAL fluid. Mice were treated with curcumin at indicated doses (ip injection 1 h after influenza A viruses (IAV) infection once daily for consecutive 4 days). BAL fluid was obtained at day 4 post‐infection. Immune cell profiles were characterized by the numbers of total cells (A), macrophages (B), neutrophils (C), and lymphocytes (D). Data are mean±SD *P<.05, **P<.01, ***P<.001 vs control (first column); #*P*<.05, ##*P*<.01 vs veh group (second column)

To determine whether curcumin affects immune cells, mice were exposed to IAV for 1 hour and then treated with curcumin once daily for consecutive 4 days. On day 4 post‐infection, mice BAL fluid was obtained, and immune cell profiles were characterized by counting the immune cell numbers. The fluorescence‐activated cell sorting (FACS) results showed that IAV infection induced significant expanding of total cells, macrophages, neutrophils, and lymphocytes. However, curcumin could inhibit the proliferation of these immune cells in a dose‐dependent manner (Figure 4A‐D).

**Figure 5:**
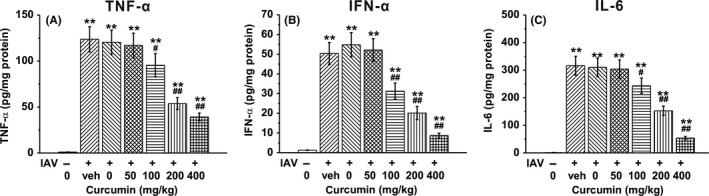
Effects of curcumin treatment on cytokine production in BAL fluid, including TNF‐α (A), IFN‐α (B) and IL‐6 (C). Mice were treated with curcumin at indicated doses (ip injection 1 hour after influenza A viruses (IAV) infection once daily for consecutive 4 days). BAL fluid was obtained at day 4 post‐infection. Levels of cytokines were quantified by corresponding enzyme‐linked immunosorbent assay (ELISA) assays. Data are mean±SD **P<.01 vs control (first column); #*P*<.05, ##*P*<.01 vs veh group (second column)

To demonstrate the effect of curcumin on mice BAL fluids, we detected the secretion of the pro‐inflammatory cytokines TNF‐α, IFN‐α, and IL‐6, which contribute to IAV‐induced lung inflammation. TNF‐α, IFN‐α, and IL‐6 levels were significantly elevated in the IAV‐infected groups compared with the control group, which received no IAV infection. However, treatment of curcumin dramatically reduced the secretion of TNF‐α, IFN‐α, and IL‐6 in a dose‐dependent manner (Figure 5A‐C).

## 4 DISCUSSION

The remittent explosion of pandemic influenza and appearance of novel IAV is worldwide threat. Some of the infection results in severe pneumonia or death, although majority of patients present with mild symptoms. Great efforts have been made to investigate the mechanism of pathogenesis of IAV‐induced diseases, and certain vaccines have been clinically utilized worldwide. However, the morbidity and mortality keep high.^28^ IAV infection is one of the reasons causing acute lung injury (ALI), which promotes the intravascular activation of inflammatory cells, and ultimately causes the injury of endothelial or parenchymal cells.^29^ As early as in 1995, Rogy and colleagues have demonstrated that the overexpression of TNF‐α receptor efficiently protected mice from LPS‐induced sepsis. As high levels of pro‐inflammatory cytokines play an essential role in inflammatory response, strategies to inhibit their functions or promote the impact of anti‐inflammatory cytokines have been investigated.^30^

Our present work reveals that upon the IAV infection, human macrophages produced significantly higher levels of pro‐inflammatory cytokines, such as TNF‐α, IFN‐α, and IL‐6, compared to that with no virus infection. The dysregulation of pro‐inflammatory cytokine production induced by IAV was further confirmed *in vivo* by infection of mice with IAV. The results are consistent with many other studies that upon the infection of IAV, mice produced dramatically higher levels of TNF‐α, IFN‐α, and IL‐6.^30^, ^31^ Thus, we aimed to find out an agent to inhibit the inflammatory responses of macrophages and alleviate the acute lung injury induced by IAV infection.

Curcumin is the major curcuminoid derived from turmeric, which is a member of the ginger family. It exhibits anti‐inflammatory, anti‐tumor, anti‐oxidative activities, as well as safety.^32^, ^33^ The diverse bioactivities of curcumin derive from its capability to modulate a number of signaling pathway components, such as transforming growth factor‐β, phosphorylase kinase, adhesion molecules, 5‐lipoxygenase (5‐LOX), C‐reactive protein, STAT3, pro‐inflammatory cytokines, NF‐κB, and apoptotic proteins. Moreover, different formulations of curcumin have been investigated, such as powder, tablets, capsules, emulsions, liposomal encapsulation, and even nanoparticles. In fact, in clinical trials, curcumin has been utilized alone or in cooperation with other drugs.^33^

In our present study, we used curcumin of different concentration to treat the IAV‐infected human macrophages and found that curcumin could neither significantly affect the viability of IAV‐infected human macrophages, nor obviously impact the percentage of virus‐infected macrophages. This indicated that curcumin has no effect on the virus‐defense ability of human macrophage, as well as the viability of these innate immune cells. However, by examining the culture medium, we found that curcumin indeed downregulate the pro‐inflammatory cytokine (TNF‐α, IFN‐α and IL‐6) production of IAV‐infected human macrophages.

We further established the *in vivo* mouse model by exposing the mice with IAV via intratracheal route, and then treated the mice with curcumin of certain concentrations. We tested the cytokine contents in BAL fluid and found that IAV infection resulted in the outbreak of pro‐inflammatory cytokine, such as TNF‐α, IFN‐α, and IL‐6. However, when the infected mice received curcumin administration, these pro‐inflammatory cytokines dramatically decreased in a dose‐dependent manner. It demonstrated the ability of curcumin to inhibit IAV‐induced infection via downregulating the pro‐inflammatory cytokine. When we examined the immune cell characteristics in the BAL fluid, we found that the immune cells, including macrophages, neutrophils, and lymphocytes, underwent an explosion upon the IAV infection, but soon decreased in numbers after the curcumin administration. Thus, we deduced that under the IAV challenge, curcumin exerted its anti‐inflammatory ability by both inhibiting the immune cell expanding and downregulating the pro‐inflammatory cytokine production.

Previous studies have revealed that NF‐κB signaling pathway is one of the most important pathways that are activated in macrophages under the pathogen challenge.^34^, ^35^, ^36^, ^37^, ^38^ Thus, we aimed to study whether the anti‐inflammatory ability of curcumin is associated with NF‐κB signaling pathway. By examining the protein expression levels via Western Blotting, we found that IAV infection leads to the decrease in IκBα and cytosolic p65, while enhanced the levels of p‐IκBα and nuclear p65. However, administration of curcumin exactly reversed this phenomenon. IκBα is a NF‐κB inhibitor that could be phosphorylated and degraded when cells suffering IAV challenge. P65 is a component of NF‐κB heterodimer, which is associated with NF‐κB nuclear translocation and activation.^27^ Thus, it is reasonable that when the cells are infected with IAV, IκBα underwent degradation and phosphorylation, facilitating the activation of NF‐κB. While as a component of NF‐κB heterodimer, p65 could translocate from the cytoplasm to the nuclear, to promote the expression of pro‐inflammatory‐related genes. These findings indicated that NF‐κB might be a target of curcumin to inhibit the pro‐inflammatory responses of immune cells. Our results are consistent with the anti‐atherosclerotic study of Cao et al., in which they revealed that in oxidized low‐density lipoprotein (oxLDL) stimulated macrophages, curcumin reduced the levels of CD147 and matrix metallopeptidase 9 (MMP‐9) by inhibiting the NF‐κB signaling pathways.^39^

In conclusion, we found that IAV infection could result in the inflammatory responses of immune cells, especially macrophages. Our results suggest that curcumin confers protection against IAV‐induced acute lung injury by limiting immune cell expanding and downregulating pro‐inflammatory cytokine production through inhibiting NF‐κB signaling pathway. The anti‐inflammatory effects of curcumin indicate that it has therapeutic potential for acute lung inflammatory disease.

## CONFLICTS OF INTEREST

The authors declare that they have no conflict of interest.
